# Physical fitness in institutionalized older adults with dementia: association with cognition, functional capacity and quality of life

**DOI:** 10.1007/s40520-019-01445-7

**Published:** 2020-01-11

**Authors:** A. Sampaio, I. Marques-Aleixo, A. Seabra, J. Mota, E. Marques, J. Carvalho

**Affiliations:** 1 CIAFEL-Research Center in Physical Activity, Health and Leisure, Faculty of Sport, University of Porto, Rua Dr. Plácido Costa 91, 4200-450 Porto, Portugal; 2 Faculty of Psychology, Education and Sports, Lusófona University of Porto, Rua de Augusto Rosa 24, 4000-098 Porto, Portugal; 3 Research Center in Sports Sciences, Health Sciences and Human Development, CIDESD, University Institute of Maia, ISMAI, Maia, Portugal

**Keywords:** Alzheimer’s disease, Physical fitness, ADL, Ageing, Institutionalization

## Abstract

This cross-sectional study investigated the association of physical fitness with cognitive function, functional capacity and quality of life among institutionalized older adults with dementia. One hundred and two older adults aged 78.0 ± 8.4 years, predominantly female (67.6%), with neurocognitive disorder due to Alzheimer’s disease (AD) (49.2%), vascular dementia (14.7%), Parkinson’s disease (2%), dementia with Lewy bodies (2%) or unspecified dementia (32.1%) participated in the present study. Regression analyses were used to examine associations between physical fitness components (Senior Fitness Test) and cognitive function (Mini-Mental State Examination), functional capacity (Katz Index of Independence in Activities of Daily Living) and Quality of Life (QoL)-Alzheimer's Disease scale. Univariate regression indicates that strength, flexibility, agility/dynamic balance and aerobic endurance are relevant for cognitive function, physical capacity and perceived QoL in institutionalized older people with dementia. After multiple regression analyses, adjusted for body mass index (BMI), results showed that aerobic endurance had a significant positive association with Total Katz Index. For both, caregiver perception of QoL-AD and global QoL-AD, BMI remained significantly and positively associated. Agility–dynamic balance presented a significant negative relation with global QoL-AD. Overall, our findings suggest that better physical fitness is important for cognition and autonomous functional capacity and that it has positive repercussions on the QoL in institutionalized older adults with dementia. Consequently, exercise-based therapeutic strategies aiming to improve physical fitness should be implemented.

## Introduction

Dementia is a progressive degenerative syndrome that affects memory, thinking, behaviour and the ability to perform activities of daily life (ADL) [1]. According to the Fifth Edition of the American Psychiatric Association’s Diagnostic and Statistical Manual (DSM-5), dementia is a major neurocognitive disorder (MND) and is caused by a variety of neurodegenerative diseases, including Alzheimer's disease (AD, most common type), vascular dementia, dementia with Lewy bodies and Parkinson's disease. Dementia affects over 48 million people worldwide, and it has been predicted that this number will double every 20 years [2]. Due to its progressive degenerative nature, this syndrome contributes to physical, cognitive and social disabilities that culminate in functional dependency, usually leading to institutionalization. Therefore, dementia has devastating social consequences, not least on healthcare costs, effectively making it one of the leading age-related health problems affecting worldwide [1]. Major international public health organizations and national governments are calling for policies to solve and/or mitigate the global burden of dementia [3]. Among the innumerous preventive and therapeutic strategies used to counteract dementia-associated diseases, physical exercise has been considered a promising non-pharmacological approach.

Ageing is associated with a decrease in all health-related components of physical fitness, including cardiorespiratory endurance, muscular endurance, muscular strength, body composition and flexibility [4]. Moreover, as older adults become less physically active during the ageing process [5, 6], their physical fitness is negatively affected. Physical fitness components are always required for carrying out ADL, such as getting up from a sitting or lying position, taking a shower, avoiding obstacles and walking [5, 7]. However, functional capacity, in the sense of the ability to perform ADL autonomously [8], is not only associated with physical fitness but also with psychological factors acting together over extended time [9]. Frequently, older adults with dementia live in nursing homes and other institutional settings, which lack opportunities for physical activity [10], thus exacerbating the decline of physical fitness.

Conversely, it seems that higher levels of physical activity/fitness could help reduce the incidence of dementia. In a large cohort of men and women surveyed over 17 years, having better fitness was associated with a lower risk of mortality from dementia [11]. A prospective study with 163,000 older adults found that physical activity can reduce the risk of dementia by 28% and AD by 45% [12].

Exercise interventions for people with dementia have shown positive results in the ability to perform ADLs. Based on randomized controlled trials, Forbes and collaborators [13] concluded that older adults with dementia did find exercise beneficial to their ability to perform ADL. A recent meta-analysis with ten randomized controlled trials showed a moderate-to-large positive effect after combined cognitive–physical interventions for ADL [14].

The existing body of literature reveals modest findings between fitness training and improvements in cognition. Karssemeijer and collaborators [14] reported a small-to-medium positive effect of combined cognitive–physical interventions on global cognitive function in older adults with mild cognitive impairment (MCI) or dementia. Another meta-analysis that included 39 studies has shown that physical exercise improved cognitive function in the over 50 s, regardless of the cognitive status of participants evidence [15]. However, a previous Cochrane review that examined the effect of exercise for older people with dementia revealed no clear evidence of benefit from exercise on cognitive functioning [13]. Therefore, the association between participation in physical activity programs and cognition in older adults with dementia still needs to be further investigated.

Quality of life (QoL) is a multidimensional concept, and physical fitness has been considered one of its critical components due to its importance in performing ADL independently [16]. In fact, among older adults with dementia, a negative QoL is associated with diminished physical and cognitive function [17]. As a factor that can be modified by increasing physical activity, physical fitness is essential for maintaining the motor skills that are critical for performing ADL and consequently for enhancing QoL in people with dementia [13].

Although there are a growing number of studies on exercise programs applied to adults with dementia suggesting beneficial effects, a wide variety of methodologies regarding duration (e.g. 2 weeks;18 months), frequency (e.g. twice per week; daily) and exercise type (e.g. aerobic, strength, balance) and different dementia population (stages of dementia; institutionalized or in community settings, among others) may contribute to inconsistent or even contradictory outcomes [13]. Therefore, further research is needed to develop efficient exercise programs, and it is also necessary to identify which physical fitness components are more relevant for institutionalized older adults with dementia. This study aimed to clarify the association between different physical fitness components, as a modifiable factor throughout life particularly with exercise engagement, with cognitive function, functional capacity and QoL in institutionalized older people with dementia.

## Materials and methods

### Participants and study design

The participants of this cross-sectional study were recruited in six different Portuguese nursing homes. One hundred and two older adults from both genders, aged 65–93 years and all diagnosed by a physician with an age-related neurocognitive disorder, agreed to participate in the study. The eligible subject pool was restricted to older adults with the following characteristics: age ≥ 65 years, institutionalized for more than 6 months, diagnosis of an age-related neurocognitive disorder and absence of any diagnosed or self-reported musculoskeletal or cardiovascular disorders that would contraindicate participation in physical fitness testing. During the initial screening visit, all participants, formal caregivers and institutions received a complete explanation of the purpose, risks and procedures of the study. Written informed consent was provided, and the review boards of the six nursing homes approved all methods and procedures. The investigation was in full compliance with the Helsinki Declaration [18]. The sociodemographic and clinical characteristics of participants at baseline are shown in Table 1.

**Table1:** Characteristics of the participants

	Participants (*n *= 102)
Age (years)	78.0 ± 8.4
Men, no. (%)	33 (32.4%)
Educational level (years)	3.48 ± 3.3
Neurocognitive disorder due to, no. (%)	
Alzheimer's disease	50 (49.2%)
Vascular disease	15 (14.7%)
Parkinson's disease	2 (2%)
Lewy bodies disease	2 (2%)
Unspecified	33 (32.1%)
Diagnosis (others than NCD), no. (%)	
Hypertension	32 (31.4%)
Heart disease	20 (19.6%)
Diabetes mellitus	15 (14.7%)
Osteoporosis	9 (8.8%)
Blood pressure, (mmHg)	
Systolic	125.8 ± 20.8
Diastolic	73.9 ± 12.3
Physical fitness	
Lower body strength (Rps)	9.7 ± 4.2
Upper body strength (Rps)	10.7 ± 5.0
Lower body flexibility [25]	19.2 ± 10.8
Agility/dynamic balance (sec)	19.2 ± 10.5
Upper body flexibility [25]	40.4 ± 14.2
Aerobic endurance (step)	64.5 ± 31.9
MMSE (points)	17.9 ± 4.9
Total Katz (points)	13.5 ± 4.2
Quality of life (points)	
Participant total score	25.8 ± 5.2
Caregiver total score	24.2 ± 5.6
Global total score	25.3 ± 4.6

Number and proportional distributions are presented for categorical variables: gender; neurocognitive disorders and diagnosis. All other variables are mean ± standard deviation

### Measurements

All measurements were performed by the same evaluators, in six different nursing homes. At each nursing home, the test was of 1-week duration. In the morning, participants completed anthropometric measurements (weight and height) and performed Senior Fitness Test (SFT) battery and Quality of Life-Alzheimer’s Disease scale (QoL-AD) questionnaire, while, in the afternoon, formal caregivers filled Katz Index and QoL-AD questionnaire. Despite different institutions, all assessments were organized in the same way.

### Physical fitness

Physical fitness was assessed with the SFT [7], which is considered a reliable instrument for assessing physical fitness in older adults (≥ 60 years of age), including older people with cognitive impairment [19]. Participants performed six tests: chair stand test (to assess lower body strength); arm curl test (to measure upper body strength); 2-minute step test (to assess aerobic endurance); chair sit and reach test (to assess lower body flexibility); back scratch test (to assess upper body flexibility); and 8-foot up and go test (to assess agility and dynamic balance).

### Cognitive function

The Mini-Mental State Examination (MMSE) [20] was used for a global cognitive evaluation. This instrument is clinically used to assess cognitive mental status, as well as to detect and follow the course of mental illness. It assesses orientation, attention, immediate and short-term recall, language and the ability to follow simple verbal and written instructions. A total score categorizes the individual on a scale of cognitive function ranging from 0 to 30 [20]. MMSE normative values consider the subjects’ educational level. Operational cut-off values for the Portuguese population [21] are 22 (for 0–2 years of literacy), 24 (for 3–6 years of literacy) and 27 (for more than 6 years of literacy).

### Functional capacity

A Portuguese modified version of Katz Index [22] was used to evaluate participant's functioning capacity based on caregiver report. The index includes six items: bathing, dressing, transferring, feeding, incontinence, toileting and the sum of all items to calculate the Katz total [22, 23]. Independence levels for the ADL questions are recorded on a scale of 0–4, divided in the following categories: 1 dependent; 2 independent with help; 3 independent with supervision; 4 independent [22].

### Quality of life

The QoL-AD scale [24] was used to measure the participant's QoL. The questionnaire includes 13 items: physical health, energy, mood, living situation, memory, family, marriage, friends, self as a whole, ability to do chores, ability to do things for fun, financial situation and QoL as a whole. The QoL-AD uses both self-report and the caregiver's reports of the participant's QoL and is scored on a 4-point Likert scale ranging from 1 (poor) to 4 (excellent), with total scores ranging between 13 and 52 points.

### Statistical analysis

Characteristics of the sample were expressed either as means and standard deviations or proportions. Multiple regression analyses with cognition, functional capacity and QoL as the dependent variables and physical fitness components as independent variables were used. Candidate variables for the multivariable model were screened with univariate methods. At each step, the independent variable not in the model that had the smallest p-value was entered, and variables already in the model were removed if their *p* value became larger than the significance level. The model was terminated when no more variables were eligible for inclusion or removal. Significance level in all analyses was set at 0.05. SPSS version 24.0 was used in all analyses.

## Results

The characteristics of the participants are summarized in Table 1. The 102 participants were predominantly female (67.6%) and had a neurocognitive disorder (NCD) due to AD. Hypertension, a minor heart condition, diabetes mellitus and osteoporosis were the other main diagnoses besides NCD.

**Fig. 1:**
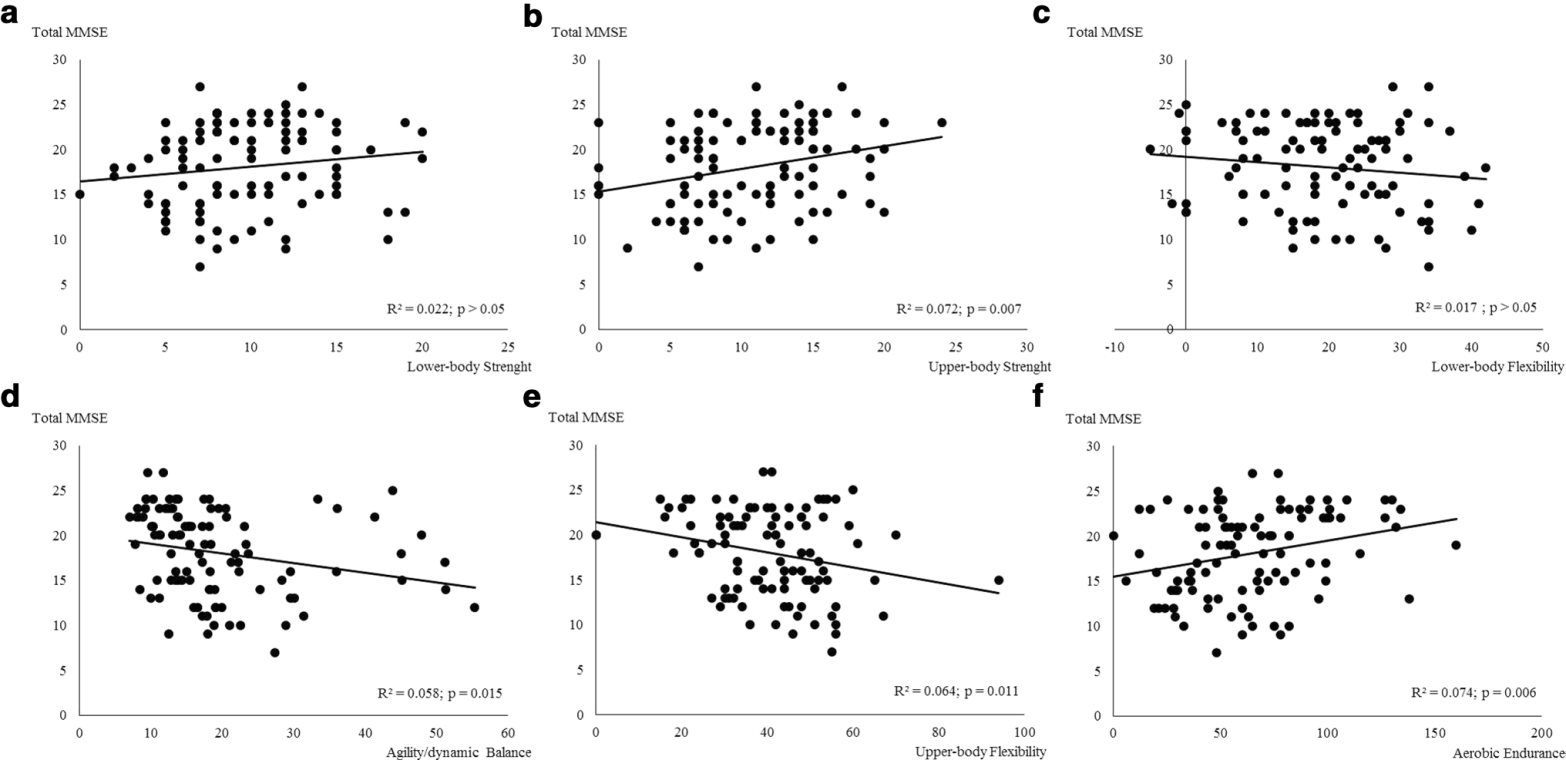
Regression relationships between Total MMSE score (points) and physical fitness components

The association between the cognition (assessed by total MMSE) and physical fitness components is presented in Fig. 1. Except for the lower body strength and flexibility (*p* > 0.05), physical fitness components were significantly associated with cognition. Aerobic endurance and upper body strength were the components with a higher relation with cognition (Fig. 1b, f), explaining ≈ 7.4% and 7.2% of the variance, respectively.

**Fig. 2:**
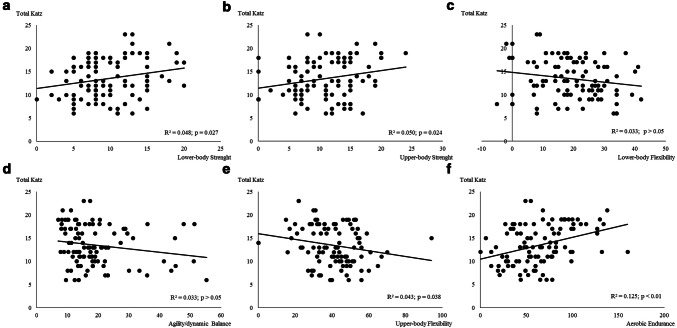
Regression relationships between functional capacity (total Katz, points) and physical fitness components

The association between functional capacity (assessed by total Katz) and physical fitness components is presented in Fig. 2. Lower body flexibility and agility and dynamic balance were not associated with cognition (*p* > 0.05). All the other physical fitness components were significantly associated with functional capacity. Aerobic capacity was the component with the higher relation with functional capacity (Fig. 2f), explaining ≈ 12.5% of the variance.

**Fig. 3:**
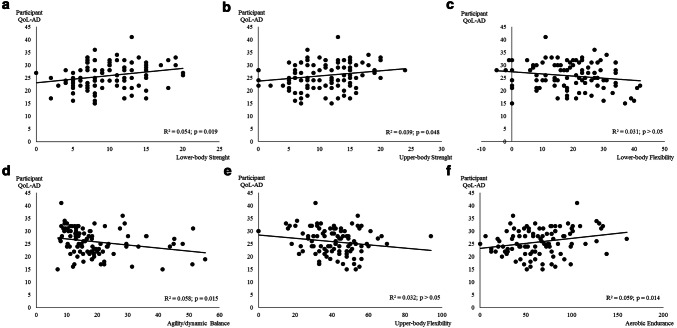
Regression relationships between quality of life perceived by the participant (participant QoL-AD, points) and physical fitness components

**Fig. 4:**
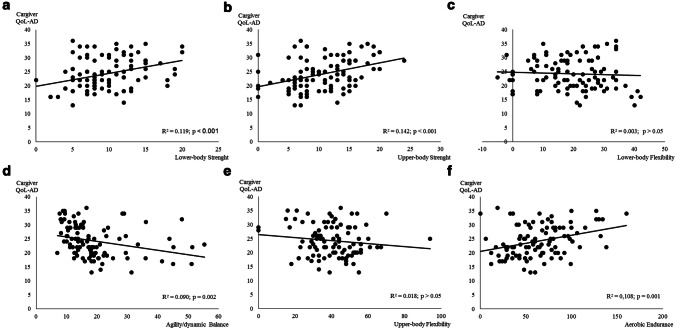
Regression relationships between the quality of life of the participant perceived by the caregiver (Caregiver QoL-AD, points) and physical fitness components

The association between QoL perceived by the participant and physical fitness components is presented in Fig. 3. Flexibility (upper and lower body) was not significantly associated with QoL (*p* > 0.05). All the other physical fitness components were significantly associated with QoL perceived by the participant. Aerobic endurance and agility/dynamic balance were the components with the higher relation with QoL perceived by the participant (Fig. 4f, d), explaining ≈ 5.9% and 5.8% of the variance, respectively.

The association between QoL perceived by the caregiver and physical fitness components is presented in Fig. 4. Flexibility (upper and lower body) was not associated with QoL perceived by the caregiver (*p* > 0.05). All the other physical fitness components were significantly associated with QoL perceived by the participant. Lower and upper body strength was the components with the higher relation with QoL perceived by the participant (Fig. 4a, b), explaining ≈ 12% and 14% of the variance, respectively.

**Fig. 5:**
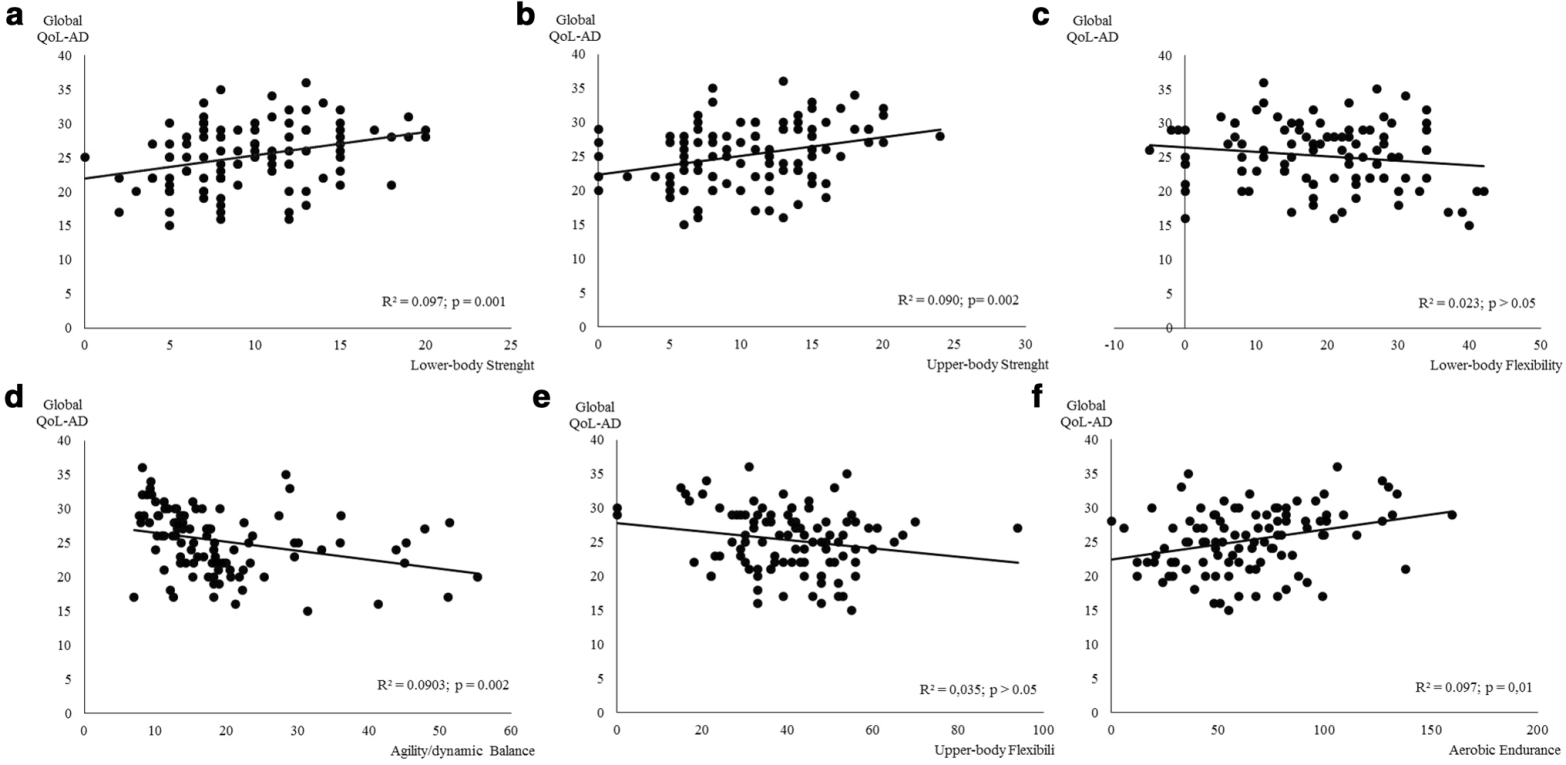
Regression relationships between global quality of life of the participant (perception of participant + perception of the caregiver) (global QoL-AD, points) and physical fitness components

The association between global QoL and physical fitness components is presented in Fig. 5. Flexibility (upper and lower body) was not associated with QoL perceived by the caregiver (*p* > 0.05). All the other physical fitness components were significantly associated with QoL perceived by the participant. Lower body strength and aerobic endurance were the components with a higher relation with global QoL (Fig. 5a, f), explaining ≈ 9.7% of the variance for both components.

Adjusted coefficients and 95% CI with cognition, functional capacity and quality of life as the dependent variables in linear multiple regression analyses are presented in Table 2. Age, gender and educational level were not correlated with cognition, functional capacity or QoL (data not shown); thus, they were not included as confounding variables in the regression models. As BMI was associated with global QoL and caregiver´s perception of QoL (data not shown), this variable was included as confounders variable in the multiple regression models for global QoL and caregiver´s perception of QoL. Aerobic endurance had a significant positive association with total Katz. For both caregiver perceptions of QoL-AD and global QoL-AD, BMI remained significantly and positively associated. Agility–dynamic balance presented a significant negative relation with global QoL-AD. Additionally, lower body strength had a significant positive effect on caregiver QoL-AD. Neither component of physical fitness was associated with the variables cognition nor was participant perception of QoL, suggesting that for these variables, there is no physical fitness component that stood out above the others.

**Table 2:** Multiple linear regression analyses of associations between the cognition, functional capacity and quality of life and the components of physical fitness

Multiple linear regression	Adjusted *R*^2^	Adjusted β (95% CI)	*p* value
Cognition^a^			
Functional capacity^b^			
Aerobic endurance	0.097	0.04 (0.01–0.08)	0.012
Participant QoL-AD^c^			
Caregiver QoL-AD^d^			
BMI	0.346	0.42 (0.15–0.70)	0.003
Lower body strength		0.51 (0.03–0.98)	0.038
Global QoL-AD^d^			
BMI	0.295	0.31 (0.09–0.54)	0.008
Agility/dynamic balance		− 0.18 (− 0.30–0.06)	0.003

^a^Multiple regression adjusted for upper body strength, agility/dynamic balance, upper body flexibility and aerobic endurance

^b^Multiple regression adjusted for lower and upper body strength, upper body flexibility and aerobic endurance

^c^Multiple regression adjusted for lower and upper body strength, agility/dynamic balance and aerobic endurance

^d^Multiple regression adjusted for lower and upper body strength, agility/dynamic balance, aerobic endurance and BMI

## Discussion

Although the relationship between higher levels of physical activity and functional capacity has been established previously [25, 26], the association between the specific dimensions of the physical fitness and the ability to perform ADL are still unclear, particularly among older adults with dementia. Moreover, studies regarding the impact of physical fitness on cognition mainly investigate aerobic fitness [13] while the association between different components of physical fitness and cognitive function needs to be clarified in this population. Overall, this study demonstrates that different components of physical fitness are relevant for cognitive function and functional capacity in institutionalized older people with dementia. As physical fitness can modulate the cognitive function and functional capacity and as these are important for QoL [13], this study also highlights the association between different physical fitness components and the QoL in this particular population.

### Strength

The prevalence of age-related loss of muscle mass and strength (sarcopenia) seems to be more predominant in older adults with dementia than older adults without dementia [27]. Gillette-Guyonne et al. [28] reported a sarcopenia prevalence of 40.6% in female subjects with AD in comparison with 21.9% in age-matched controls with no AD. Although the mechanisms behind these differences are not completely clear, dementia and sarcopenia may share certain mechanisms of pathogenesis [27, 29], such as malnutrition, hormonal changes, oxidative stress, inflammatory process and usually involving a decreased exercise and activity [30]. Previous reports suggested that the unintended weight loss and decreased BMI [31] observed in older adults with AD were predominantly related to the loss of lean mass (i.e. sarcopenia) [32]. In our study, multivariate linear regression showed that BMI was positively associated with caregivers’ perception and with global QoL, and thus, it is possible to speculate that the amount of lean mass influences the QoL in older adults with dementia. Sarcopenia has been suggested to mediate the association between poor cognition and functional decline in cognitively impaired older adults [29]. Nourhashemi [33] and collaborators found that low cognitive function was associated with muscle loss in a group of over 7,000 community-dwelling older women. Raji [34] and collaborators showed an association between having poor cognition and lower handgrip muscle strength through a 7-year cross-sectional study, with community-dwelling older adults. Accordingly, in our study, upper body strength showed itself as positively associated with cognitive function (Fig. 1) in institutionalized older adults with dementia.

Deterioration of functional capacity, in general, represents an increased risk of frailty, loss of independence and physical disability [35, 36]. In agreement with the literature, our data have shown that upper and lower body strength was significantly associated with functional capacity and QoL (Figs. 2, 3, 4, 5). Although the functional capacity and QoL are generally considered to be directly connected, in the present study we observed that only lower body strength, in the multivariate model, was still positively associated with QoL from the caregivers’ perspective (Table 2). Since lower body strength is crucial to perform daily tasks (e.g. bathing, transferring or dressing) with a higher degree of autonomy, we might speculate that the caregivers related it with a better QoL.

### Flexibility

Articular mobility may reduce across the age span [36] and can compromise functional capacity, as well as contributing towards the loss of autonomy and independence [37, 38]. This could be even more problematic in institutionalized older adults with dementia. The range of motion of the upper limb joints has been considered imperative for the ability to perform ADLs [38]. Additionally, decreased flexibility of the lower limbs has been associated with the risk of injuries and falls and changes in the gait pattern [37]. Although the present study failed to find an association between lower body flexibility and functional capacity, a significant association between upper body flexibility with cognition and functional capacity was found in this frail population. This can suggest that some ADL (such as bathing and dressing) performed autonomously, requires upper body flexibility and may be cognitively challenging for people with dementia. Unexpectedly, no significant results were observed between flexibility and QoL.

### Agility/dynamic balance

Our results showed that better agility/dynamic balance scores were positively associated with general cognition. Older adults that have a better agility/dynamic balance also have more opportunities for physical practice, more autonomy and better environmental interaction, which could be important for maintaining their cognitive function. Evidence shows that cognition plays a key role in the regulation and control of mobility [39]. This motor behaviour involves dissociable neural systems which control gait initiation, planning and execution and the adaptation of these movements to meet motivational and environmental demands [40]. Cross-sectional studies have shown that the level of cognition is related to mobility, in community-dwelling older adults [41–44]. Agility/dynamic balance is inversely associated with the risk of falls [37, 45] being one of the main geriatric problems that significantly increase the risk of hospitalization and institutionalization [46], consequently decreasing their QoL. In our study, agility/dynamic balance scores were positively associated with participants’ perception, caregivers’ perception and global QoL. After a multivariate linear regression, agility/dynamic balance was still positively associated with global QoL. These results corroborate the literature that had already established the link between mobility and QoL, in older adults without dementia [47]. Furthermore, Davis et al. [48] pointed to agility/dynamic balance as a predictor of health-related QoL in community-dwelling older adults.

### Aerobic endurance

Aerobic endurance is the most frequently studied component of physical fitness in older adults with dementia, being considered a predictor of cognitive performance [49], in healthy older adults [50] and the most effective type of exercise to improve cognition in older adults with dementia [13]. Our study results show a positive association between aerobic endurance with cognition, corroborating other studies’ results that have shown that higher levels of aerobic fitness are related to better brain health [51–53] and cognition [54], preserving critical brain areas in cognitively healthy older adults [51] and persons with AD [55], as well as reduced brain atrophy in those with early-stage AD [56]. Multiple mechanisms may account for associations between aerobic endurance and cognitive function. Higher aerobic fitness is linked with upregulation of neurotrophins (e.g. the brain-derived neurotrophic factor, BDNF), neurovascular plasticity (primarily via angiogenesis) and neurogenesis contributing to better cerebral health [57, 58]. BDNF levels (which contribute to growth regulation, maintenance and survival of neurons) can be increased by aerobic endurance training [57, 59, 60]. In our study, aerobic endurance is also associated with functional capacity. After a multivariate linear regression, aerobic endurance is still positively associated with functional capacity. These results are according to the literature [61–63] that have shown aerobic endurance capacity as a physical fitness component that is directly and independently associated with the ability to perform ADL independently.

## Conclusion

In summary, we believe that our findings contribute to a better understanding of the impact of different physical fitness components on cognition, functional capacity and QoL in institutionalized older adults with dementia. Although aerobic endurance stands out as the key factor of physical fitness in association with cognitive function, functional capacity and QoL, the results of this study suggest that the contribution of every component of physical fitness is singular and irreplaceable. Thus, strategies to attenuate the decline of different physical fitness components, such as the implementation of multicomponent exercise interventions (combined aerobic exercise and flexibility or strength and agility/balance training programs), may contribute to preserve cognitive function, functional capacity and to maintain QoL, in this particular population.

## Data Availability

The data that support the findings of this study are available from the corresponding author upon reasonable request.
